# Gas Sensing Properties of Epitaxial LaBaCo_2_O_5.5+δ_ Thin Films

**DOI:** 10.1038/srep10784

**Published:** 2015-07-06

**Authors:** M. Liu, S. P. Ren, R. Y. Zhang, Z. Y. Xue, C. R. Ma, M. L. Yin, X. Xu, S. Y. Bao, C. L. Chen

**Affiliations:** 1 Electronic Materials Research Laboratory, Key Laboratory of the Ministry of Education & International Center for Dielectric Research, Xi’an Jiaotong University, Xi’an 710049, People’s Republic of China; 2 State Key Laboratory for Mechanical Behavior of Materials, Xi’an Jiaotong University, Xi’an 710049, People’s Republic of China; 3 School of Science, Xi’an Technological University, Xi’an 710032, P. R. China; 4 Department of Physics and Astronomy, University of Texas at San Antonio, TX 78249, United States; 5 The Texas Center for Superconductivity, University of Houston, Houston, Texas 77204, United States

## Abstract

Chemical reactivity and stability of highly epitaxial mixed-conductive LaBaCo_2_O_5.5+δ_ (LBCO) thin films on (001) LaAlO_3_ (LAO) single-crystalline substrates, fabricated by using pulsed laser deposition system, were systematically investigated. Microstructure studies from x-ray diffraction indicate that the films are *c*-axis oriented with the interface relationship of [100]_LBCO_//[100]_LAO_ and (001)_LBCO_//(001)_LAO_. LBCO thin films can detect the ethanol vapor concentration as low as 10ppm and the response of LBCO thin film to various ethanol vapor concentrations is very reliable and reproducible with the switch between air and ethanol vapor. Moreover, the fast response of the LBCO thin film, as the *p*-type gas sensor, is better than some *n*-type oxide semiconductor thin films and comparable with some nanorods and nanowires. These findings indicate that the LBCO thin films have great potential for the development of gas sensors in reducing/oxidizing environments.

Cobalt-based oxides have attracted scientists and engineers much attention due to their unique magnetic, electrical transport and optical properties, which have been used to design various new concept devices, such as solid state fuel cell, energy harvest, gas sensors, etc^1^,^2^,^3^,^4^,^5^,^6^,^7^,^8^,^9^,^10^. Among them, the oxygen-deficient double perovskite cobaltates, LaBaCo_2_O_5.5+δ_ (LBCO), show particularly interesting phenomena with the A-site order, nanoscale order, and disorder structures, such as, different spin state configurations, giant MR effect, etc^11^,^12^,^13^,^14^,^15^,^16^. To under these physics nature, single crystalline LBCO thin films are required. Recently, highly epitaxial single crystalline LBCO thin films were fabricated on various substrates, such as (001) LaAlO_3_ (LAO)^16^, (001) SrTiO_3_^17^,^18^,^19^, (001) MgO^20^,^21^, and (110) NdGaO_3_^22^. The LBCO films not only exhibit a much larger magnetoresistance value than those from various phases of its bulk material, but also possess an extraordinary sensitivity to H_2_ and O_2_, especially an exceedingly fast redox reaction at high temperature and a superfast oxygen vacancy exchange diffusion chemical dynamics^16^,^23^. Therefore, the LBCO thin films are one promising candidate for the development of the gas sensors in reducing/oxidizing environments, which is one of the critical research issues in the public safety and energy security.

Gas-sensing materials using the *n*-type oxide semiconductor have been intensively studied, such as ZnO^24^,^25^, TiO_2_^26^,^27^,^28^, SnO^29^,^30^,^31^, WO_3_^32^, Fe_2_O_3_^33^,^34^,^35^, In_2_O_3_^36^, *etc*. However, there are very few reports on the gas-sensing materials using *p*-type oxide semiconductor, such as NiO^37^,^38^,^39^, CuO^40^, LaFeO_3_^41^, Co_3_O_4_,^42^,^43^,^44^,^45^,^46^,^47^,^48^,^49^,^50^,^51^,^52^,^53^,^54^,^55^ LBCO^16^,^23^. Compared with n-type oxide semiconductor for gas-sensing, the p-type oxide semiconductors with distinctive surface reactivity and oxygen adsorption can enhance gas selectivity, decrease the dependence of humidity to negligible level and improve the recovery speed, reviewed by Kim and Lee^46^. Also, it is a good catalyst to promote selective oxidation of various volatile organic compounds^47^,^48^,^49^,^50^.

Various oxidation states (Co^2+^ /Co^3+^ /Co^4+^) of cobalt in LBCO thin film make it to be a good candidate as *p*-type gas sensor. The conductivity is strongly dependent on the valence states, which can change the LBCO from good conductive to insulate. Ideally, when the valence states are all Co^+3^, the LBCO should be insulate. And when the valence states are mixed with Co^+3^ and Co^+4^ or Co^+2^ and Co^+3^, the LBCO should be semiconductor or semimetallic, respectively. Since the LBCO films were grown in the pure oxygen of 250 mTorr, the films are good conductive. Moreover, the Co^+4^ is not as stable as Co^+3^. Therefore, the valence states should be a mixed state of Co^+3^ and Co^+4^. When LBCO thin film is exposed to the reducing gas, parts of Co^+4^ changed into Co^+3^ and the resistance increases. In the contrast, the gas change to oxidizing gas, parts of Co^+3^ changed into Co^+4^ and the resistance decreases^51^,^52^. Ethanol vapor is safer and more active than other gas, such as H_2_, ammonia, hydrogen sulfide, which is used as the representative of reducing gases. In this paper, we explore here the transient responses of the LBCO thin films to various ethanol vapor concentrations at different operating temperatures. It was found that even though ethanol vapor concentration is as low as 10 ppm, the LBCO thin film still can detect the transient response. Moreover, the response time can be reduced to ~24 s under the ethanol vapor concentration of 400 ppm with the recovery time of only ~10 s at 375 ^o^C. These results indicate that the LBCO thin films have great potential for the development of the gas sensor applications in reducing/oxidizing environments, expect the applications on giant magnetoresistance and cathode for solid oxide fuel cell.

## Results and Discussion

**Figure 1:**
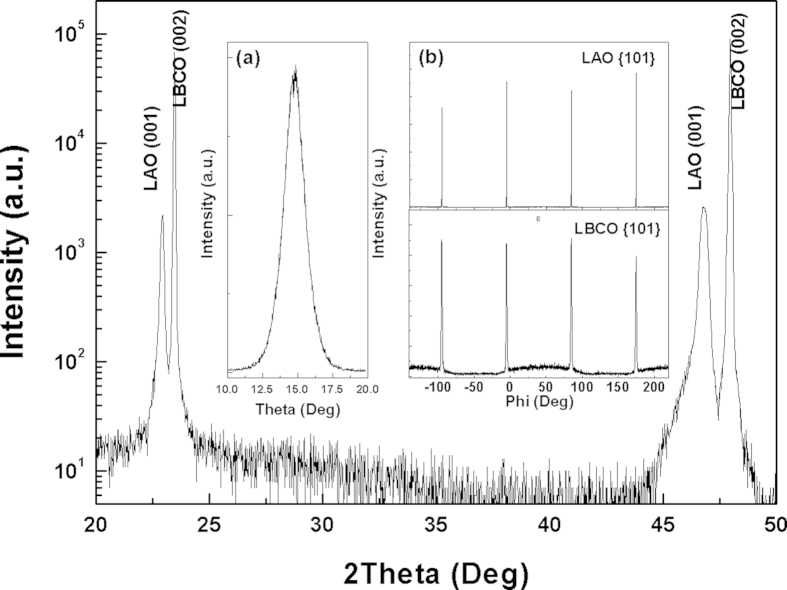
Typical XRD pattern of the LBCO films grown on (001) LAO substrates. The inset is (**a**) rocking curve from the (002) reflection for the LBCO film and (**b**) the φ scans taken around the {101} diffraction of the LBCO film and LAO substrate.

**Figure 2:**
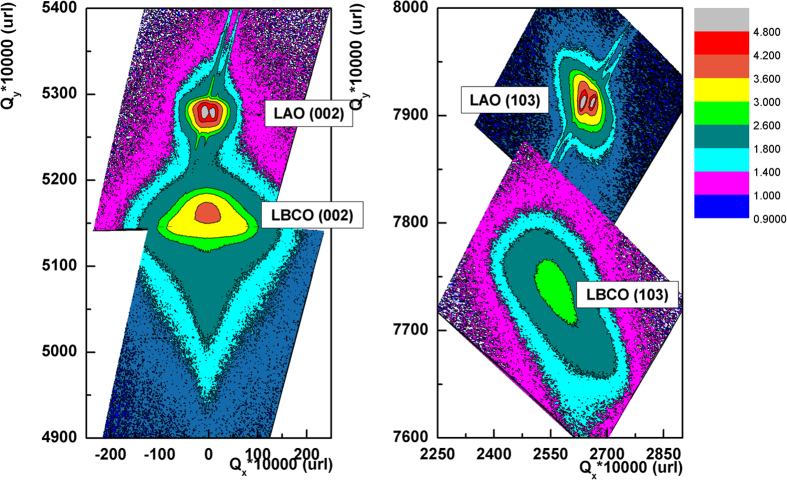
Reciprocal space mappings taken around from the symmetric (002) and asymmetric (103) reflections of the LBCO films and LAO substrates.

The crystalline quality of the LBCO thin films was characterized by the XRD *θ−2θ* scan, rocking curve, φ scan and reciprocal space mappings (RSMs). Figure 1 is a typical *θ−2θ* pattern for the LBCO thin films on (001) LAO substrates showing that only the (00*l*) peaks can be detected for the LBCO thin films. It is revealed that the films are *c*-axis oriented or the *c*-axis normal to the substrate surface. The rocking curve measurements from the (002) reflections for the LBCO films show that the Full Width of Half Maximum (FWHM) is ~0.92°, as shown in the inset (a) of Fig. 1, indicating that the LBCO films on LAO substrates have good crystalline quality. To understand the in-plane interface relationships between the LBCO films and the LAO substrates, the φ scans measurements have been performed. The inset (b) of Fig. 1 is the φ scans taken around from the {101} reflections of the LBCO films and LAO substrates. The four-fold symmetry and sharp peaks in the φ scan suggest that the films have good epitaxial nature. Therefore, the orientation relationships between the thin films and the substrates can be determined to be [100]_LBCO_//[100]_LAO_ and (001)_LBCO_//(001)_LAO_. RSMs is a very effective method to study the microstructure information, such as lattice parameters, defect density, domain structure, etc. in order to obtain the lattice parameters and interface strain of LBCO films on LAO substrates, the RSMs have been performed taken around from the symmetric (002) and asymmetric (103) reflections of the LBCO films and LAO substrates, as shown in the Fig. 2. According to the Bragg law and the angles’ relationship between these crystalline planes, the in-plane (*a* & *b*) abd out-of–plane (*c*) lattice parameters of LBCO films on LAO substrates can be calculated from the RSMs. The in-plane and out-of-plane lattice parameter is 3.90 Å and 3.88 Å, respectively. It is shown that this LBCO film on LAO is full relaxation state.

**Figure 3:**
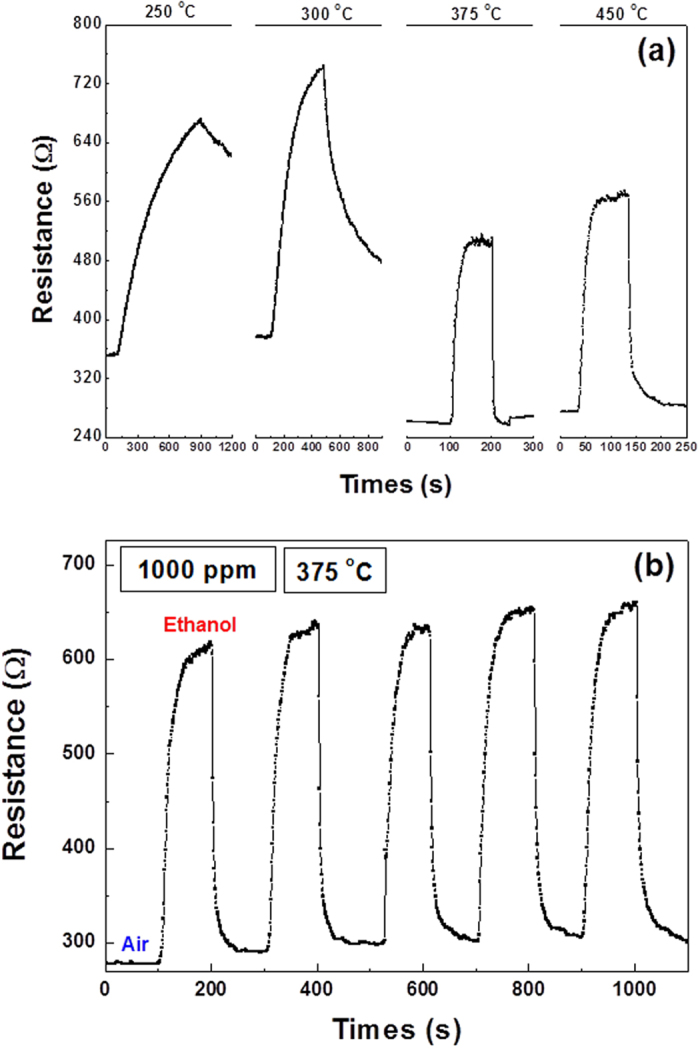
(**a**) Transient responses at different temperatures from 250 to 450 °C, (**b**) transient response vs. number of exposure tests during the change of air to 1000 ppm ethanol vapor concentration at 375 ^o^C.

In order to identify the optimum working temperature for the LBCO gas sensors, the sensing transient response of the LBCO films to 1000 ppm ethanol vapor exposure were investigated as a function of sensor temperature from 250 ^o^C to 450 ^o^C, as shown in the Fig. 3(a). It is clearly seen that the resistance of LBCO thin film increase when it is exposed to the ethanol vapor (reducing gas), then the resistance decrease when the gas change from 1000 ppm ethanol vapor to air, which is in agreement with our previous studies under the gas switch between H_2_ and O_2_^16^,^23^. In fact, the LBCO thin films will generate a high density of oxygen vacancies when it is exposed to the reducing gas^53^, such as ethanol vapor gas. This chemical processing can be represented by:
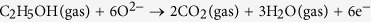


The electrons are released into the LBCO films through the oxidation reactions with ethanol vapor reducing gas. Thus, the increased electronic density will result in the increase of the resistance due to the LBCO films are p-type semiconductors, On the other hand, the resistances of the LBCO thin films will decrease when exposed to oxidizing gas, such as air. The response time from air to 1000 ppm ethanol vapor decrease with the temperature increase. Especially, the response time rapidly drop and become faster when the temperature exceed to 375 ^o^C. Although the LBCO thin films can detect ethanol gas at the temperature of 250 ^o^C and 300 ^o^C, the resistance of LBCO thin film can’t recovery completely in short time when it exposed to air. Thus, the working temperature above 300 ^°^C is better for the excellent performance as ethanol/reducing gas sensor. Meanwhile, the stability of the LBCO thin film as ethanol/reducing gas sensor has been examined, as shown in Fig. 3(b). The response and recovery repeatability of the LBCO thin film is examined under 1000 ppm ethanol vapor at 375 °C. It shows that the resistance change of LBCO thin film can keep its initial value after circle operation, revealing that the LBCO thin film as ethanol/reducing gas sensor have good reproducibility.

**Figure 4:**
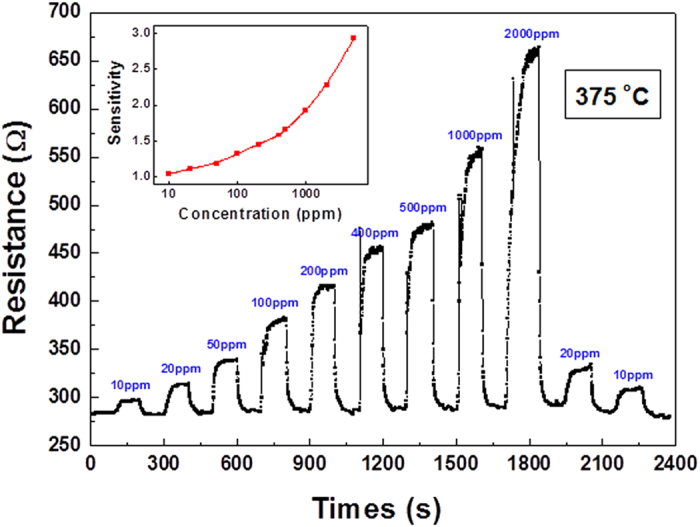
Transient responses and the sensitivity (inset) of the LBCO gas sensors during the change of air to various ethanol vapor concentrations at 375 °C.

To investigate the sensitivity of LBCO thin film as ethanol gas sensor, the transient response or the resistance switching behavior during the change between air and various ethanol vapor concentrations at 375 ^o^C was examined. As shown in the Fig. 4, the LBCO thin films have reliable and reproducible responses during the change between air and various ethanol vapor concentrations. Even at very low concentration of 10 ppm, the LBCO films still can detect transient response. The ratio of resistance switching between air to ethanol vapor is strongly dependent upon the ethanol vapor concentrations, or linearly-like increases with the increase of the ethanol vapor partial pressures from 10 ppm to 5000 ppm, as seen in the inset of Fig. 4. The sensitivity (*S*) of the LBCO thin film as the ethanol gas sensor increase form 1.04 at 10 ppm to 2.92 at 5000 ppm. The *S* determined by using the formula *S* = R_g_/R_a_, where R_a_ and R_g_ are resistance of the gas sensor in air and in testing gas atmosphere, respectively. The sensitivity can be enhanced by replacing the air with oxygen gas.

**Figure 5:**
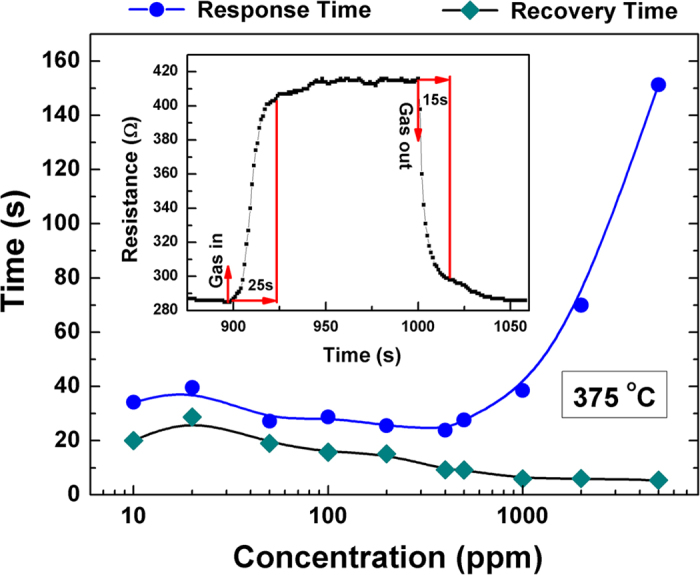
The plot of response time and recovery time of the LBCO gas sensors during the change of air to various ethanol vapor concentrations at 375 °C, the inset shows the definition of the response and recovery times.

It is known that the response and recovery times are the two important quantity factors for the gas sensors, As shown in the inset of Fig. 5, the response time is the time taken by the resistance change from R_air_ to R_air_+[R_gas_-R_air_]*90%, and the recovery time is the time taken by the resistance change from R_gas_ to R_gas_– [R_gas_-R_air_]*90%. The response and recovery factors for the LBCO thin films as ethanol gas sensors are shown in Fig. 5 and Table 1. The response time decreases from ~34 s (10 ppm) to ~24 s (400 ppm) with the increase of ethanol vapor concentrations. Then, the response time increases with the increase of the ethanol vapor partial pressures from 500 ppm to 2000 ppm. Therefore, the optimum response time is ~24 s at the concentration of 400 ppm. The reason for the phenomena is still unclear and will be explored in the following work. There is no transition for the recovery time, which decreases from ~29 s (10 ppm) to ~6 s (2000 ppm) with the increase of the ethanol vapor concentration. Compared with some ethanol sensors, epitaxial LBCO thin film exhibits better quality factors. Table 2 gives the as-reported response times of various ethanol sensors at intermediate temperature ranges. Obviously, the response (recovery) time of the LBCO epitaxial thin films is less than that from the *n*-type gas sensors based on the NiO thin films, SnO_2_ thin film and TiO_2_ thick films. Normally, the response of *p*-type oxide semiconductor gas sensor is slower than *n*-type semiconductor gas sensor^54^. Moreover, it is comparable with the ZnO nanorods, CuO microsphere, hollow sea urchin-like structure *α*-Fe_2_O_3_ and various nanostructure Co_3_O_4_ listed in the Table 2. Nanorods and nanowires normally have fast response due to the large reactivity area. The comparable fast response in LBCO thin films indicates that epitaxial LBCO thin films will be an good gas sensor for reducing gas, especially for ethanol vapor. The response and recovery time definitely can be improved by adjusting the nanostructure of LBCO, like LBCO nanorods and nanowires. Therefore, LBCO materials have great potential for the development of the application of gas sensors in reducing/oxidizing environments.

In summary, the sensing transient response of the epitaxial LBCO thin films during the changes of air to various ethanol vapor exposure have been investigated in the temperature range of 250 ^o^C to 450 ^o^C. It is shown that the LBCO thin films have reliable and reproducible responses to ethanol vapor, even at very low concentration of 10 ppm. The optimum response time is ~24 s under the ethanol vapor concentration of 400 ppm with the recovery time of ~10 s at 375 ^o^C. The performance of epitaxial LBCO thin film as the ethanol vapor sensor is better than that of some *n*-type oxide semiconductor thin films and comparable to that of the nanorods and nanowires structure. It is revealing that the LBCO thin films can be a promising candidate as gas sensor in reducing/oxidizing environments.

## Methods

### Film preparation

A KrF excimer pulsed laser deposition system with a wavelength 248 nm was used to fabricate the LBCO thin films on (001) LAO single-crystalline substrates. Details of the processing conditions can be found in the related literatures^17^. Briefly, the films were grown under an oxygen pressure of 250 mTorr at 700 °C with the laser energy density of about 2.0 J /cm^2^ at 10 Hz. The typical film thickness is about ~200 nm with the growth rate of ~13.3 nm/min. After growth the films were annealed at 700 ^o^C for 15 min in pure oxygen (200 Torr) and then cooled down to room temperature at the rate of 30 ^o^C/min.

### Structural and electrical characterization

The crystalline quality and epitaxial behavior of the LBCO thin films were characterized by high-resolution x-ray diffraction (HRXRD) using PANalytical X’Per MRD. The LBCO thin films have been used for the gas sensing measurements. The ethanol sensing properties of the LBCO thin films were systematically studied using the GS-1TP intelligent gas sensing analysis system (Beijing Elite Tech. Co. Ltd., China)^55^. This analysis system consists of several parts: a heating system, gas distribution system, probe system, vacuum system, measurement system and related control software. The samples can be heated up to 500 °C with a precision of 1 °C. In order to obtain the accurate data, all the samples will be preheated at the working temperature for ~20 min. Once the LBCO gas sensors were stable, the ethanol gas will be injected into the test chamber. After the resistance of the LBCO gas sensor reaches a new constant value, we will open the test chamber to recover LBCO sensor into the air atmosphere.

## Additional Information

**How to cite this article**: Liu, M. *et al.* Gas Sensing Properties of Epitaxial LaBaCo_2_O_5.5+δ_ Thin Films. *Sci. Rep.* **5**, 10784; doi: 10.1038/srep10784 (2015).

## Figures and Tables

**Table 1:** Sensitivity, response time and recovery time for the LBCO film gas sensors under during the change of air to various ethanol vapor concentrations at 375 ^o^C.

**Ethanol vapor concentrations(ppm)**	**Sensitivity**	**Response time(s)**	**Recovery time(s)**
10	1.04	34	20
20	1.11	40	29
50	1.19	27	19
100	1.33	29	16
200	1.45	26	15
400	1.58	24	9
500	1.65	28	9
1000	1.92	38	6
2000	2.28	70	6
5000	2.92	151	5.5

**Table 2:** Comparison of Various Oxide Semiconductors in Ethanol Vapor Sensing.

**Materials**	**Ethanol Concentration(ppm)**	**Working Temperature(^o^C)**	**Response Time(s)**	**Recovery Time(s)**	**Reference**
LBCO Thin Films	100	375	29	16	This paper
Co_3_O_4_ Nanofibers	100	336	22.7	2.4	43
Co_3_O_4_: Nanosheets, Nanorods, Nanocubes	100	400	66, 29, 49	10–13	45
*α*-Fe_2_O_3_ Hollow Sea Urchin-like Structures	21–2100	350	7–21	11–14	35
CuO Microsphere	200	400	9–17	4–17	40
NiO: Thin Films, Hollow Hemispheres	100	300	62, 49	47, 42	39
ZnO Nanorods	100	370	22	27	25
TiO_2_ Thick Films	300	350	128	348	28
SnO_2_ Thin Film	1250	350	42	88	31
